# Biomechanical and physiological effects of a passive upper-body exoskeleton during stair ascent and descent

**DOI:** 10.1371/journal.pone.0343438

**Published:** 2026-02-20

**Authors:** Gabriela Garcia, Milena Espoz, Camilo Albuja, Rafaella Yañez, Paul G. Arauz, Bernard J. Martin

**Affiliations:** 1 Departmento de Ingeniería Industrial, Colegio de Ciencias e Ingenierías, Universidad San Francisco de Quito USFQ, Quito, Ecuador; 2 Department of Orthopaedics, Renaissance School of Medicine, Stony Brook University, Stony Brook, New York, United States of America; 3 Department of Industrial and Operations Engineering, University of Michigan, Ann Arbor, Michigan, United States of America; The Hong Kong Polytechnic University, HONG KONG

## Abstract

The market has seen the emergence of various passive exoskeletons designed to assist with carrying tasks; however, evidence of their effects during stair negotiation remains limited. In this study fifteen female and fifteen male participants carried a 12-kg load with and without an upper-body passive exoskeleton (CarrySuit^Ⓡ^) while ascending and descending stairs. The impact of the CarrySuit^Ⓡ^ was evaluated through measurements of heart rate, muscle activity, and joint range of motion during both stair ascent and descent. The results indicated that heart rate and muscle activity in the biceps brachii and erector spinae were reduced during both ascent and descent when wearing the exoskeleton, except in females during descent, where muscle activity remained comparable between conditions. An increase in upper-leg muscle activity was observed only in males during ascent, while lower-leg muscle activity was reduced during descent for all participants. Some side-based asymmetries in lower-limb activation were observed, but they were generally modest when using the exoskeleton. Use of the CarrySuit^Ⓡ^ was associated with reduced joint range of motion, particularly in males, affecting the shoulders, elbows, pelvis, hips, and thorax. In females, reductions in range of motion were limited to the neck and thorax. In contrast, increases in range of motion were observed in the ankles, knees, hips, and shoulders in females, and in the ankles and knees in males. Lower perceived discomfort was reported by all participants when using the exoskeleton, with broader relief observed among male users. These findings suggest that the CarrySuit^Ⓡ^ has a positive impact on physiological, biomechanical, and perceptual outcomes during stair-based load carrying, and may serve as a viable ergonomic solution for work environments where mechanical aids are impractical.

## Introduction

Manual material handling remains a core component of many industrial and labor-intensive sectors, despite growing trends in automation and mechanization. International reports indicate that over 30% of workers regularly carry and transport heavy loads as part of their daily tasks [1]. These physically demanding activities are closely associated with the development of musculoskeletal disorders (MSDs), particularly in the lower back, lower extremities, and upper limbs [2–5]. Work-related MSDs are among the leading causes of occupational disability, negatively impacting productivity, worker well-being, and overall economic outcomes [6–8].

Efforts to mitigate physical workload in occupational environments have led to the exploration of various ergonomic strategies. However, traditional solutions such as automation, material handling equipment, or workspace redesign are not always feasible, especially in complex or constrained environments such as stairwells, multi-story buildings, construction sites, and uneven outdoor terrain. In such settings, wearable assistive technologies, including exoskeletons, have gained attention as practical tools for alleviating physical strain and reducing the risk of injury. Passive exoskeletons, in particular, offer a promising balance of structural support and affordability by using energy-storing components or frame-based load distribution systems rather than powered actuators [9].

Although passive exoskeletons have been extensively studied for static tasks, overhead work, and lifting [10,11], only a limited number are specifically designed to support dynamic load carriage, and even fewer have been evaluated in conditions involving stair negotiation [12–14]. One such device is the CarrySuit^Ⓡ^, a commercially available passive upper-body exoskeleton designed by Auxivo AG (Schwerzenbach, Switzerland). The device redistributes load across the torso and hips, reducing strain during manual carrying tasks. Previous studies have demonstrated positive effects of the CarrySuit^Ⓡ^ during level and inclined walking, including reductions in muscle activation and heart rate [12–15]. However, its performance during stair ascent and descent remains largely unexamined.

Stair negotiation poses unique biomechanical and physiological challenges, making it a critical yet underexplored context for exoskeleton evaluation. Stair ascent and descent require substantial joint mobility, dynamic balance, and neuromuscular coordination [16,17]. When combined with the task of carrying a heavy load, these demands are significantly heightened. Unlike level or inclined walking, stair negotiation introduces alternating concentric and eccentric loading patterns in the lower limbs and alters movement strategies. Inefficient or asymmetrical load distribution in this context may result in compensatory motions and increased joint loading, potentially elevating injury risk [18,19]. Given these concerns, understanding how exoskeletons like the CarrySuit^Ⓡ^ influence movement quality, physiological strain, and symmetry during stair-based tasks is essential. Furthermore, sex-based differences in biomechanical responses and user perceptions have been documented in previous exoskeleton studies [13,14,20], underscoring the need for inclusive evaluations.

Accordingly, this study aimed to investigate the physiological and biomechanical effects of using the CarrySuit^Ⓡ^ during stair ascent and descent while carrying a 12 kg load, a condition that remains untested despite the exoskeletons’ growing adoption. This work addresses a critical gap in the literature: although the CarrySuit^Ⓡ^ has shown benefits on level and inclined surfaces, its impact on stair negotiation, a biomechanically demanding and common workplace scenario, has not been evaluated. To this end, we assessed muscle activity, sagittal plane joint kinematics, heart rate, and perceived discomfort to determine how the exoskeleton influences physical effort, movement control, and user experience during stair-based carrying. Specifically, the following research questions were addressed:

- Does carrying 12 kg with the CarrySuit^Ⓡ^ on stairs influence heart rate, discomfort, and muscle activity (erector spinae, vastus lateralis, gastrocnemius medialis, and biceps brachii) compared to performing the same task without the exoskeleton in both males and females?
- Does wearing the CarrySuit^Ⓡ^ alter sagittal plane joint kinematics during stair ascent and descent relative to the no-exoskeleton condition in both sexes?
- Are side-based asymmetries in joint angles or muscle activity affected by exoskeleton use?

By answering these questions, the present study contributes much-needed evidence on the applicability of passive upper-body exoskeletons in complex, vertical movement tasks, offering critical insights for their ergonomic implementation in occupational settings where mechanical aids are impractical.

## Methods

### Participants

The study involved thirty adults, equally divided between men and women, all of whom had no prior experience with exoskeletons. Participants were recruited via internal university announcements and social media platforms. Participants volunteered and underwent a brief screening oral questionnaire administered by the research team to confirm the absence of MSDs, physical or neurological conditions, or pregnancy that might affect their ability to carry a 12 kg load or wear the exoskeleton. The average demographic data were a weight of 66.70 ± 7.28 kg, height of 176.20 ± 6.99 cm, and age of 21.33 ± 1.36 years for males, and a weight of 59.56 ± 8.39 kg, height of 160.80 ± 4.17 cm, and age of 21.47 ± 1.25 years for females. All participants were right-handed. The tested sample size provided an achieved statistical power of 91%, based on a partial eta-squared (ηp2) effect size of 0.1, and an alpha level of 0.05 (calculated using G*Power 3.1.9.7).

The study was approved by the Ethics Committee of the Universidad San Francisco de Quito (#2021-145M) and adhered to the principles outlined in the Declaration of Helsinki. All participants provided written informed consent before the experimental sessions. Participants were recruited from August 15, 2022 until December 31, 2022. This study is part of a larger investigation [15] in which participants also tested the exoskeleton during inclined treadmill walking.

### CarrySuit^Ⓡ^ exoskeleton

**Fig 1:**
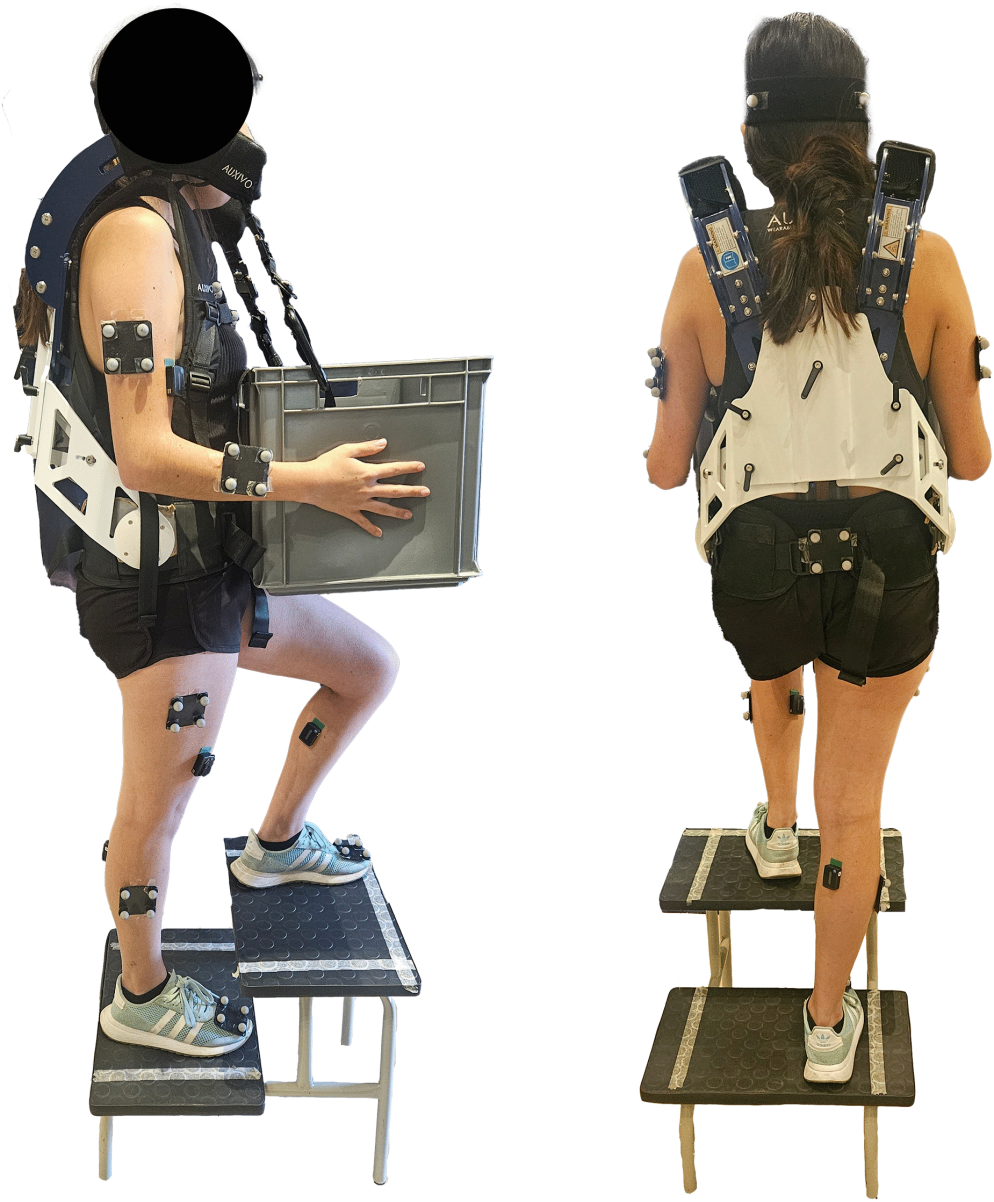
Participant wearing the CarrySuit^Ⓡ^ exoskeleton (EXO condition) while ascending stairs while carrying a 12 kg load.

The carrying task was assessed both with and without the passive upper-body exoskeleton, the Auxivo CarrySuit^Ⓡ^ (v1.0, Schwerzenbach, Switzerland, 2021). The CarrySuit^Ⓡ^ is designed to reduce strain on the upper extremities and back during heavy manual material handling, as reported by Auxivo. Weighing 5.6 kg, the exoskeleton supports an external load of up to 50 kg. Its design resembles a backpack, featuring textile straps and cushioned contact points that connect to the hips, chest, and shoulders. The exoskeleton includes a rigid, yet adjustable, framework that extends from the hips to the shoulders, accommodating a range of anthropometric dimensions. Proper fit was ensured by adjusting the straps and aligning the frame to each participant’s torso and hip measurements, as recommended by the manufacturer. Fit was confirmed both visually and through participant feedback prior to beginning trials. External objects were secured to the front frame using straps with carabiners (see Fig 1). The load was primarily transferred through the hips and shoulders, and, since the system lacks active energy storage mechanisms such as springs, it primarily provides support during static carrying rather than during lifting or lowering actions.

### Measures and instrumentation

#### Electromyography (EMG).

Surface bipolar Trigno EMG sensors (Delsys Inc., Boston, MA), with a sampling frequency of 1926Hz, were placed bilaterally on the biceps brachii along the line connecting the medial acromion to the cubital fossa at one-third of the distance from the cubital fossa [21]. Avanti EMG Sensors (Delsys Inc., Boston, MA), a newer model with a sampling frequency of 2148 Hz, were used for all other muscles tested. Sensors for the vastus lateralis were positioned at two-thirds of the distance along the line extending from the anterior superior iliac spine to the lateral aspect of the patella [21]. Sensors for the medial gastrocnemius were placed at the most prominent portion of the muscle belly [21]. For the erector spinae, sensors were positioned at the L3 height, approximately 3 cm to the left and right of the spine [22]. To ensure optimal contact, the skin was prepared by shaving, cleaning with alcohol, and lightly abrading with gel (Skin Prep Gel, Nuprep®, Aurora, USA).

Data processing was performed in MATLAB (MathWorks, Inc., Natick, USA), including detrending, filtering with a 4th-order Butterworth bandpass filter (30–300 Hz), and Fast Fourier Transform for visual inspection of the power spectra to ensure the absence of noise. Root mean square (RMS) values were calculated using a 250 ms moving window with 50% overlap and normalized to the average of the peak EMG values obtained from three submaximal voluntary contractions per muscle. These peak values were obtained during isometric exercises described in the procedure section [23]. The RMS normalization was then applied to the EMG signals recorded during the experimental stair trials. The primary outcome variables were the mean and peak normalized EMG amplitudes (% RMS values) for each muscle. The mean normalized EMG amplitude reflects the average level of muscle activation throughout the task [24], whereas the peak normalized EMG amplitude, defined as the 90th percentile of the normalized values, represents the highest consistently sustained muscle activity during the task [25].

#### Kinematics.

**Fig 2:**
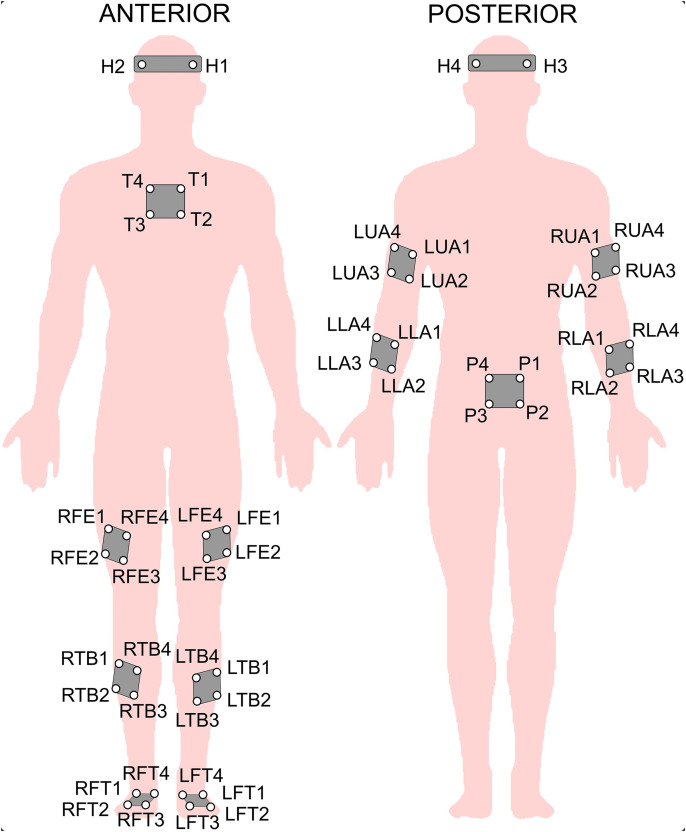
Reflective marker placement for 3D motion capture. Abbreviations: L = Left, R = Right; FE = femur, TB = tibia, FT = foot, LA = lower arm, UA = upper arm, H = head, T = thorax, P = pelvis.

A 10-camera motion capture system (Vicon MX, Oxford, UK) recorded whole-body kinematics at a sampling frequency of 100 Hz. Groups of four 10 mm reflective spherical markers, as well as individual markers, were placed on anatomical landmarks including the head, thorax, pelvis, humerus, radius, femur, tibia, and foot (as depicted in Fig 2) to define their local coordinate systems. Following the methodology of previous studies [14,26,27], a biomechanical model including 52 markers was employed to obtain comprehensive whole-body kinematic data. 3D joint angles were calculated for the head-trunk, pelvis-trunk, shoulder (humerus-thorax), elbow (humerus-radius), hip (pelvis-femur), knee (femur-tibia), and ankle (foot-tibia) relative to a neutral standing posture used as the zero reference. The Cardan angle sequence [28] was used to determine sagittal plane angles.

Data were exported and processed in MATLAB by a custom program. Angular data were segmented into individual strides, and a time-normalized waveform (0–100%) of the typical stair gait cycle was created using 1% sample steps [26,27,29]. Normalized strides for the leading leg were defined as beginning with the pull-up of the leading leg and heel contact on the first stair, followed by weight acceptance, push-off, pull-up of the stance leg, forward movement to the second stair, foot clearance, and ending with the placement of the leading leg on the second step (S1 Fig). Three cycles were averaged for each leading side, and the range of motion (ROM) was calculated for each joint angle.

#### Heart rate.

Heart rate, measured in beats per minute (bpm), was assessed before and during the carrying task using a chest belt heart rate monitor (Polar H10, Polar, Finland) with a 1000 Hz sampling frequency. At the start of each experimental session, participants rested in an armchair for 5 minutes, and their resting heart rate was recorded, noting the lowest value observed. Heart rate was also continuously monitored throughout the carrying task, and the average heart rate for all stair trials was calculated.

#### Subjective evaluation of discomfort and fatigue.

To assess participants’ subjective experiences, a two-part questionnaire was administered both before and after each carrying task. The first section focused on discomfort perception and was adapted from the Nordic Musculoskeletal Questionnaire [30], as in previous studies [31–33]. Participants were presented with a human body diagram featuring horizontal visual analogue scales (VAS) for the neck, shoulders, arms, hands/wrists, back, upper legs/hips, knees, lower legs, ankles, and feet. The 10 cm VASs were anchored with “no discomfort” on the far left (0 mm) and “extreme discomfort” on the far right (10 cm). Participants marked the scale to indicate their perceived level of discomfort, and the results were recorded in centimeters. A similar VAS was included to assess overall fatigue. The second section of the questionnaire explored the perceived usability and acceptability of the exoskeleton. It included four questions prompting participants to rate: (1) how easy was the task to perform while wearing the exoskeleton, (2) how comfortable the task felt, (3) whether they preferred performing the task with or without the exoskeleton, and (4) whether they would recommend the exoskeleton for similar carrying tasks.

### Procedure

Upon arrival of the participant at the laboratory, a demonstration of the exoskeleton was given after a brief introduction. Written informed consent was obtained, and demographic data including age, sex, and handedness were collected, while stature and body mass were measured using a weight scale and a stadiometer, respectively. A 5-minutes rest was then provided while the resting heart rate was measured. Afterward, EMG electrodes were placed on the selected muscles, and participants performed three repetitions of 10-second isometric submaximal voluntary contractions for each muscle, with 1-minute rest periods in between.

For the biceps brachii, elbow flexion exercises in the vertical plane were performed with a 7.5 kg kettlebell [34]. For the gastrocnemius medialis, calf raises were performed in the upright standing posture while holding a 7.5 kg kettlebell [35]. For the vastus lateralis, knee extensions and leg raises with 2.5 kg ankle weight [36] were performed in a sitting posture. For the erector spinae, the Biering-Sorensen maneuver, extension of the trunk against a 5 kg resistance was performed with the whole body in a horizontal position [37]. These exercises are commonly used to obtain reference contractions for expressing muscle activity as a percentage of submaximal effort.

After the placement of reflective markers, participants first completed a 1-minute walk to warm up and become familiar with the exoskeleton. This was followed by 1-minute inclined treadmill walking trial under two conditions, with and without the exoskeleton, while carrying a 12 kg box, as part of the prior experiment reported elsewhere [15]. This sequence was chosen to provide progressive full-body familiarization with the device under dynamic but controlled conditions, allowing participants to acclimate to the exoskeleton’s support and movement constraints before performing the more demanding stair negotiation task. Afterward, a 30-minute rest period was provided. During this time, participants were seated and monitored to ensure full recovery between tasks, and rest durations were strictly timed using a stopwatch. Following the rest, participants performed a 1-minute stair ascent and descent trial to rewarm and complete initial subjective evaluations of discomfort and fatigue. These pre-task ratings were used to confirm that any subsequent discomfort reflected the experimental condition and not residual effects from previous tasks. Subsequently, the stair-carrying task with a 12 kg box was completed under two randomized conditions (with and without the exoskeleton). Randomization was performed using a simple random number generator to determine the order of conditions for each participant. The carrying task consisted of a total of six stair ascent and descent trials for each condition. One leg was randomly assigned as the lead for the first three trials (both ascent and descent), and the opposite leg was used as the lead for the remaining three trials. Stair ascent and descent were performed at a self-selected but consistent pace across conditions. Participants were instructed to maintain a natural rhythm and avoid sudden or exaggerated steps. Throughout the stair task, heart rate was monitored continuously, and kinematic and EMG data were recorded for each trial and were averaged for each condition and leading side. For symmetry comparisons, only the gait cycle data from the leading sides were analyzed.

Subjective evaluations of discomfort and fatigue were evaluated immediately after completion of each carrying condition (with and without the exoskeleton). The conditions were separated by a 10-minute rest period. After each rest, a control evaluation of discomfort and fatigue was conducted to ensure that discomfort levels had returned to baseline, confirming that subsequent ratings reflected the experimental condition rather than residual fatigue. Finally, the user satisfaction questionnaire was completed after all experimental trials. Participants were not informed of the specific study hypotheses prior to data collection to minimize potential bias in subjective responses.

### Data analysis

Statistical analyses were performed using SAS Studio (SAS Institute Inc.) with a significance level set at α = .05. Mixed models with a variance-components covariance structure and residual maximum likelihood estimation were used (via the PROC MIXED procedure in SAS) to evaluate the outcome variables: mean and peak muscular activity, heart rate, and ROM joint angles in the sagittal plane; all data fulfilled the normality tests. For muscular activity and ROM outcome variables, participants were treated as random effects, condition (with [EXO] or without [NOEXO] the exoskeleton), and side (left or right) were treated as fixed effects. For heart rate and discomfort, the statistical model included only condition as a fixed factor. Separate models were run for male and female participants to account for anatomical and biomechanical differences known to affect musculoskeletal responses, postural strategies, and exoskeleton fit and interaction. All models were run by sex. Post-hoc analyses were conducted using least square means, and p-values were adjusted using the Tukey-Kramer method. The effect size, expressed as ηp², was calculated using the Tippey & Longnecker approach for mixed models in SAS Studio [38]. Cohen’s thresholds, which categorize effect sizes as small (ηp² = .01), medium (ηp² = .06), and large (ηp² = .14), were applied to interpret the ηp² values [39]. Descriptive results are reported as the mean with standard error. As the discomfort ratings violated the assumption of normality, a nonparametric Friedman two-way analysis of variance by ranks was conducted to assess differences across conditions.

## Results

### Muscle activity

Descriptive statistics (means and standard errors) and mixed-model fixed effects results for bilateral mean and peak muscle activation during stair descent while carrying a 12 kg box, with and without the exoskeleton, are presented in Table 1 for both male and female participants.

**Table 1:** Descriptive statistics (mean and standard error) and fixed effects analysis results for bilateral mean and peak muscle activation (% normalized RMS EMG amplitude) in male and female participants during stair descent while carrying a 12 kg box, performed with (EXO) and without (NOEXO) the exoskeleton.

		Stair Descent						
Females		Mean(SE)				Fixed Effects: *p*-value (ηp²)		
		EXO		NOEXO				
		Left	Right	Left	Right	Condition	Side	Condition x Side
**Mean normalized EMG amplitude (%)**	Biceps Brachii	18.11(2.05)	33.23(3.96)	88.96(3.83)	107.19(5.70)	<.0001*(.76)	<.0001*(.14)	.47(.001)
	Erector Spinae	37.1(1.73)	41.09(2.02)	39.9(2.54)	42.48(2.51)	.07(.01)	.02*(.03)	.56(.001)
	Gastrocnemius Medialis	46.98(2.4)	55.86(2.68)	43.94(1.76)	56.44(3.29)	.52(.002)	<.0001*(.16)	.34(.01)
	Vastus Lateralis	49.18(2.5)	51.56(3.06)	49.78(2.78)	51.69(2.71)	.92(.0001)	.18(.01)	.87(.0001)
**Peak normalized EMG amplitude (%)**	Biceps Brachii	34.22(4.21)	71.91(8.28)	119.57(5.89)	133.42(6.68)	<.0001*(.55)	<.0001*(.15)	.07(.01)
	Erector Spinae	57.92(2.28)	68.91(3.49)	62.79(3.64)	67.02(3.63)	.22(.01)	.001*(.05)	.14(.01)
	Gastrocnemius Medialis	100.05(4.96)	110.48(5.21)	101.52(4.56)	131.53(7.45)	.007*(.04)	<.0001*(.11)	.02*(.03)
	Vastus Lateralis	125.42(6.87)	128.63(8.37)	120.92(7.72)	123.65(7.9)	.30(.01)	.46(.002)	.82(.0002)
**Males**		**Mean(SE)**				**Fixed Effects: *p*-value (ηp²)**		
		**EXO**		**NOEXO**				
		**Left**	**Right**	**Left**	**Right**	**Condition**	**Side**	**Condition x Side**
**Mean normalized EMG amplitude (%)**	Biceps Brachii	14.05(2.17)	16.26(2.52)	96.91(7.49)	111.28(8.29)	<.0001*(.73)	.04*(.04)	.17(.02)
	Erector Spinae	24.64(2.14)	25.52(2.57)	29.1(2.33)	33.8(2.28)	<.0001*(.2)	.02*(.02)	.54(.002)
	Gastrocnemius Medialis	39.53(2.36)	44.33(3.53)	46.84(2.59)	52.35(3.75)	<.0001*(.1)	.001*(.05)	.66(.001)
	Vastus Lateralis	42.22(4.44)	48.52(3.98)	42.51(4.44)	47.64(3.63)	.88(.0001)	.0009*(.06)	.22(.01)
**Peak normalized EMG amplitude (%)**	Biceps Brachii	27.04(4.06)	31.22(4.98)	127.65(10.65)	151.06(12.03)	<.0001*(.67)	.03*(.02)	.17(.01)
	Erector Spinae	40.62(2.91)	44.52(3.59)	50.57(3.65)	56.82(3.92)	<.0001*(.2)	.02*(.03)	.51(.002)
	Gastrocnemius Medialis	83.26(4.18)	102.67(8.81)	98.19(4.8)	115.81(8.05)	.001*(.05)	<.0001*(.09)	.98(.00001)
	Vastus Lateralis	96.87(9.09)	116.76(7.96)	101.93(9.88)	103.22(6.5)	.22(.01)	.01*(.03)	.01*(.03)

Note. Bold font and * indicate statistically significant values, α = .05. Abbreviations: EXO = with exoskeleton; NOEXO = without exoskeleton; EMG = electromyography; SE = standard error.

In females, a significant condition-by-side interaction was found only for the peak activity of the gastrocnemius medialis, with a small to medium effect size (Table 1). Post hoc comparisons revealed significant asymmetry in both conditions, with higher activation of the right gastrocnemius medialis compared to the left (EXO: *p* = .01; NOEXO: *p* = .02). Additionally, a main effect of condition was observed for the peak activity of the gastrocnemius medialis (medium effect size), and for the mean and peak activity of the biceps brachii (large effect sizes), with higher activation in the NOEXO condition compared to the EXO condition. A main effect of side was also detected for all muscles except the vastus lateralis, indicating a right-side dominance regardless of condition (Table 1).

In males, a significant condition-by-side interaction was found only for the peak activity of the vastus lateralis, with a small to medium effect size (Table 1). Post hoc comparisons revealed only one significant asymmetry: greater vastus lateralis peak activation in the right muscle compared to the left during the EXO condition (*p* = .003). Additionally, a main effect of condition was observed for the mean and peak activity of the biceps brachii, erector spinae, and gastrocnemius medialis, with higher activations in the NOEXO condition compared to the EXO condition. A main effect of side was also detected for all muscles, indicating right-side dominance regardless of condition (Table 1).

Descriptive statistics (means and standard errors) and mixed-model fixed effects results for bilateral mean and peak muscle activation during stair ascent while carrying a 12 kg box, with and without the exoskeleton, are presented in Table 2 for both male and female participants.

**Table 2:** Descriptive statistics (mean and standard error) and fixed effects analysis results for bilateral mean and peak muscle activation (% normalized RMS EMG amplitude) in male and female participants during stair ascent while carrying a 12 kg box, performed with (EXO) and without (NOEXO) the exoskeleton.

		Stair Ascent						
Females		Mean(SE)				Fixed Effects: *p*-value (ηp²)		
		EXO		NOEXO				
		Left	Right	Left	Right	Condition	Side	Condition x Side
**Mean normalized EMG amplitude (%)**	Biceps Brachii	43.37(3.94)	63.62(5.39)	118.57(6.32)	128.99(6.34)	<.0001*(.56)	.0006*(.06)	.45(.003)
	Erector Spinae	43.05(2.39)	46.85(2.26)	50.01(2.22)	46.63(2.34)	.003*(.05)	.45(.003)	.04*(.02)
	Gastrocnemius Medialis	69.88(2.88)	83.7(3.41)	69.56(2.24)	73.43(3.92)	.06(.02)	.0005*(.07)	.07(.02)
	Vastus Lateralis	66.23(3.83)	68.34(3.53)	65.16(3.47)	63.7(2.6)	.12(.01)	.79(.0003)	.41(.004)
**Peak normalized EMG amplitude (%)**	Biceps Brachii	97.55(9.96)	143.59(11.16)	173.49(10.23)	177.52(9.1)	<.0001*(.21)	.001*(.06)	.03*(.02)
	Erector Spinae	78.36(4.2)	84.6(4.11)	88.01(3.76)	84.91(3.26)	.004*(.04)	.14(.01)	.09(.01)
	Gastrocnemius Medialis	188.15(9.48)	214.55(8.85)	182.47(7.88)	202.31(11.75)	.21(.01)	.001*(.05)	.62(.001)
	Vastus Lateralis	168.08(9.22)	162.33(8.4)	153.06(8.84)	155.4(8.07)	.06(.02)	.67(.001)	.56(.002)
**Males**		**Mean(SE)**				**Fixed Effects: *p*-value (ηp²)**		
		**EXO**		**NOEXO**				
		**Left**	**Right**	**Left**	**Right**	**Condition**	**Side**	**Condition x Side**
**Mean normalized EMG amplitude (%)**	Biceps Brachii	36.02(5.13)	30.49(4.87)	113.98(8.89)	134.56(9.68)	<.0001*(.69)	.17(.01)	.01*(.04)
	Erector Spinae	28.22(2.55)	28.55(3.07)	32.06(1.88)	39.4(2.97)	<.0001*(.28)	.19(.01)	.31(.01)
	Gastrocnemius Medialis	50.23(2.57)	57.56(4.03)	49.02(2.58)	63.03(4.76)	.32(.01)	<.0001*(.12)	.13(.01)
	Vastus Lateralis	51.01(3.89)	59.76(4.73)	49.19(3.93)	56.95(4.18)	.03*(.02)	.008*(.04)	.37(.004)
**Peak normalized EMG amplitude (%)**	Biceps Brachii	81.34(11.58)	67.62(10.76)	173.87(13.89)	201.16(14.51)	<.0001*(.54)	.52(.002)	0.02*(.03)
	Erector Spinae	50.28(3.79)	54.68(5.42)	54.28(2.71)	68.79(4.93)	<.0001*(.14)	.02*(.03)	.28(.01)
	Gastrocnemius Medialis	133.33(7.9)	156.38(12.67)	135.61(7.34)	159.31(10.5)	.68(.001)	.0004*(.07)	.96(.0001)
	Vastus Lateralis	147.17(10.87)	165.62(12.04)	130.7(10.75)	145.04(9.71)	.002*(.07)	.08(.01)	.65(.001)

Note. Bold font and * indicate significant values, α = .05. Abbreviations: EXO = with exoskeleton; NOEXO = without exoskeleton; EMG = electromyography; SE = standard error.

In females, a significant condition-by-side interaction was found for the mean activity of the erector spinae and the peak activity of the biceps brachii, both showing small effect sizes (Table 2). Post hoc comparisons revealed only one significant asymmetry: during the EXO condition, the right biceps brachii exhibited higher activation compared to the left (*p* = .01), while no significant asymmetry was observed for the erector spinae or in the NOEXO condition. Additionally, a main effect of condition was identified for both the mean and peak activities of the biceps brachii and erector spinae, with medium to large effect sizes, showing higher activation levels in the NOEXO condition compared to the EXO condition. A main effect of side was also detected for the biceps brachii and gastrocnemius medialis, indicating right-side dominance regardless of condition.

In males, a significant condition-by-side interaction was observed only for the mean and peak activities of the biceps brachii, with small to medium effect sizes (Table 2). However, post hoc comparisons did not reveal any significant asymmetries in these muscles. A main effect of condition was observed for the mean and peak activities of the biceps brachii and erector spinae, with higher activation in the NOEXO condition compared to the EXO condition. Additionally, a main effect of condition was found for the mean and peak activities of the vastus lateralis, showing lower activation in the NOEXO condition compared to the EXO condition. Finally, a main effect of side was detected for the mean activation of the vastus lateralis, peak activation of the erector spinae, and both mean and peak activations of the gastrocnemius medialis, all indicating right-side dominance regardless of condition.

### Whole-body kinematics

Descriptive statistics (means and standard errors) and mixed-model fixed effects results for bilateral sagittal plane joint angles during stair descent while carrying a 12 kg box, with and without the exoskeleton, are presented in Table 3 for both male and female participants.

**Table 3:** Descriptive statistics (mean and standard error in degrees) and fixed effects analysis results for bilateral sagittal plane joint angles in male and female participants during stair descent while carrying a 12 kg box, performed with (EXO) and without (NOEXO) the exoskeleton.

	Stair Descent						
	ROM Degrees Mean(SE)				Fixed Effects: *p*-value (ηp²)		
Females	EXO		NOEXO				
Joint	Left	Right	Left	Right	Condition	Side	Condition x Side
Ankles	79.76(2.16)	79.88(1.81)	79.25(2.03)	78.94(1.65)	.69(.002)	.85(.001)	.79(.001)
Knees	98.94(1.67)	96.46(2.04)	95.30(1.92)	92.21(1.77)	.01*(.11)	.07(.05)	.83(.001)
Hips	40.58(1.24)	37.08(2.09)	34.98(1.62)	35.39(1.56)	.02*(.1)	.33(.01)	.22(.02)
Shoulders	7.80(0.53)	9.32(1.43)	8.00(0.73)	7.99(0.61)	.51(.01)	.38(.01)	.37(.01)
Elbows	8.72(1.64)	6.32(0.73)	9.08(1.07)	8.36(0.85)	.19(.02)	.10(.04)	.36(.01)
Pelvis	9.11(0.71)	9.87(0.85)	10.61(0.85)	10.95(1.21)	.11(.04)	.48(.01)	.78(.001)
Thorax	6.47(0.68)	8.44(1.46)	9.24(0.64)	8.66(0.65)	.09(.05)	.43(.01)	.16(.03)
Neck	17.05(1.46)	20.36(2.11)	22.46(2.01)	23.91(2.01)	.003*(.14)	.11(.04)	.52(.01)
**Males**	**ROM Degrees Mean(SE)**				**Fixed Effects: *p*-value (ηp²)**		
	**EXO**		**NOEXO**				
**Joint**	**Left**	**Right**	**Left**	**Right**	**Condition**	**Side**	**Condition x Side**
Ankles	72.14(2.14)	74.17(2.84)	72.83(2.97)	72.33(2.65)	.73(.001)	.59(.004)	.42(.01)
Knees	88.68(1.61)	88.78(1.84)	88.88(2.33)	86.41(1.68)	.53(.01)	.48(.01)	.44(.01)
Hips	37.75(1.76)	35.12(2.26)	34.81(1.95)	35.17(1.51)	.36(.01)	.47(.01)	.34(.01)
Shoulders	6.43(0.86)	6.06(0.47)	9.21(1.81)	10.24(1.53)	.005*(.13)	.74(.001)	.56(.01)
Elbows	5.52(1.51)	3.76(0.25)	9.98(1.67)	8.36(1.22)	.001*(.18)	.21(.02)	.99(.00001)
Pelvis	8.43(1.13)	7.68(0.79)	9.50(1.03)	9.68(0.88)	.01*(.11)	.63(.003)	.43(.01)
Thorax	7.21(0.84)	8.24(1.15)	6.85(0.49)	6.90(0.47)	.21(.02)	.43(.01)	.47(.01)
Neck	20.01(2.59)	20.53(3.53)	24.74(3.31)	22.79(2.86)	.31(.01)	.54(.01)	.34(.01)

Note. Bold font and * indicate significant values, α = .05. Abbreviations: EXO = with exoskeleton; NOEXO = without exoskeleton; ROM = range of motion; SE = standard error.

In females, a significant main effect of condition, with large effect sizes, was found for the knees, hips, and neck. Specifically, the EXO condition resulted in greater ROM in the knees and hips, and reduced ROM in the neck compared to the NOEXO condition. In males, a significant main effect of condition, also with large effect sizes, was observed for the shoulders, elbows, and pelvis, with higher ROM in the NOEXO condition compared to the EXO condition. No significant main effect of side or condition-by-side interaction was detected in either males or females.

Descriptive statistics (means and standard errors) and mixed-model fixed effects results for bilateral sagittal plane joint angles during stair ascent while carrying a 12 kg box, with and without the exoskeleton, are presented in Table 4 for both male and female participants.

**Table 4:** Descriptive statistics (mean and standard error in degrees) and fixed effects analysis results for bilateral sagittal plane joint angles in male and female participants during stair ascent while carrying a 12 kg box, performed with (EXO) and without (NOEXO) the exoskeleton.

	Stair Ascent						
	ROM Degrees Mean(SE)				Fixed Effects: *p*-value (ηp²)		
Females	EXO		NOEXO				
Joint	Left	Right	Left	Right	Condition	Side	Condition x Side
Ankles	51.43(2.04)	54.77(1.73)	48.99(1.62)	49.84(1.27)	.002*(.15)	.07(.05)	.27(.02)
Knees	96.20(2.76)	105.69(2.41)	93.94(2.46)	103.06(2.25)	.11(.04)	<.0001*(.39)	.91(.0002)
Hips	67.91(1.23)	73.45(1.63)	66.91(1.57)	69.04(1.4)	.02*(.1)	.001*(.16)	.14(.03)
Shoulders	17.01(1.54)	14.40(1.44)	13.87(1.22)	14.10(1.25)	.03*(.1)	.13(.03)	.08(.05)
Elbows	14.09(1.68)	15.08(2.19)	15.43(1.01)	16.32(1.18)	.41(.01)	.54(.01)	.98(.00001)
Pelvis	9.93(0.81)	9.29(0.46)	9.97(0.87)	10.14(0.55)	.41(.01)	.66(.003)	.45(.01)
Thorax	8.16(0.33)	8.42(0.59)	11.56(0.80)	10.26(0.97)	<.0001*(.25)	.44(.01)	.23(.02)
Neck	20.59(1.34)	20.92(1.73)	27.01(1.82)	23.91(1.76)	.0001*(.18)	.29(.02)	.19(.02)
**Males**	**ROM Degrees Mean(SE)**				**Fixed Effects: *p*-value (ηp²)**		
	**EXO**		**NOEXO**				
**Joint**	**Left**	**Right**	**Left**	**Right**	**Condition**	**Side**	**Condition x Side**
Ankles	51.64(1.92)	50.87(2.01)	48.17(1.85)	49.93(2.15)	.04*(.07)	.63(.003)	.23(.02)
Knees	93.79(2.30)	98.76(2.09)	87.26(2.46)	98.89(1.79)	.02*(.08)	<.0001*(.38)	.02*(.09)
Hips	58.27(1.53)	60.09(1.21)	60.79(1.65)	64.45(1.02)	.003*(.14)	.01*(.1)	.41(.01)
Shoulders	10.62(1.37)	9.67(1.55)	15.98(2.07)	16.71(1.73)	.0003*(.21)	.94(.0001)	.59(.004)
Elbows	7.75(0.92)	6.57(0.86)	15.16(2.21)	17.95(1.93)	<.0001*(.39)	.66(.003)	.17(.03)
Pelvis	10.52(0.91)	9.57(0.89)	12.83(1.05)	12.76(1.15)	.0001*(.19)	.49(.01)	.56(.01)
Thorax	7.02(0.67)	6.98(0.47)	8.61(0.59)	7.90(0.73)	.02*(.08)	.46(.01)	.53(.01)
Neck	19.11(1.98)	19.84(2.61)	23.42(1.94)	22.57(2.41)	.07(.05)	.98(.00001)	.63(.003)

Note. Bold font and * indicate significant values, α = .05. Abbreviations: EXO = with exoskeleton; NOEXO = without exoskeleton; ROM = range of motion; SE = standard error.

In females, a significant main effect of condition, with large effect sizes, was found for the ankles, hips, shoulders, and thorax. Specifically, ROM was greater in the ankles, hips, and shoulders, and reduced in the thorax in the EXO compared to the NOEXO condition. Additionally, a main effect of side was observed for the knees and hips, indicating greater ROM on the right side regardless of condition. However, no significant condition-by-side interaction was detected.

In males, a significant condition-by-side interaction, with medium effect size, was observed only for the knees. Post hoc comparisons revealed only one significant asymmetry: during the NOEXO condition, the right knee exhibited higher ROM compared to the left (*p* < .0001), whereas no significant asymmetry was found under the EXO condition. Additionally, a main effect of condition, with medium to large effect sizes, was observed for the ankles, knees, hips, shoulders, elbows, pelvis, and thorax. Specifically, ROM was greater in the ankles and knees, and reduced in the hips, shoulders, elbows, pelvis, and thorax in the EXO compared to the NOEXO condition. Finally, a main effect of side was observed for the knees and hips indicating greater ROM on the right side regardless of condition.

### Subjective evaluation of discomfort and fatigue

Descriptive statistics (means and standard errors) and Friedman test results for discomfort and fatigue ratings following stair ascent and descent while carrying a 12 kg box, with and without the exoskeleton, are presented in Table 5 for both male and female participants.

**Table 5:** Descriptive statistics (mean and standard error) and Friedman test results for discomfort and fatigue ratings (0-10) in male and female participants after stair ascent and descent while carrying a 12 kg box, performed with (EXO) and without (NOEXO) the exoskeleton.

	Mean(SE) ratings range 0–10		
Females	EXO	NOEXO	*p*-value
Neck	2.32(0.81)	1.55(0.77)	0.81
Shoulders	2.76(0.83)	2.22(0.84)	0.17
Upper Back	2.46(0.74)	2.05(0.82)	0.63
Elbows/Arms	0.76(0.31)	3.03(0.84)	0.11
Lower Back	0.95(0.33)	1.64(0.67)	0.60
Hands/Wrists	0.5(0.16)	3.11(0.87)	.01*
Hips	0.46(0.15)	1.09(0.43)	0.58
Knees	0.43(0.15)	0.98(0.44)	1.00
Lower Legs	0.31(0.13)	1.24(0.66)	0.26
Ankles	0.36(0.12)	1.15(0.40)	0.21
Feet	0.36(0.14)	1.31(0.65)	0.22
Overall Fatigue	2.33(0.49)	3.67(0.64)	.04*
**Males**	**EXO**	**NOEXO**	***p*-value**
Neck	1.07(0.45)	0.85(0.32)	0.77
Shoulders	1.29(0.42)	1.81(0.46)	0.26
Upper Back	1.21(0.62)	1.31(0.46)	0.26
Elbows/Arms	0.41(0.15)	3.17(0.69)	<.0001*
Lower Back	0.44(0.13)	1.96(0.53)	.04*
Hands/Wrists	0.18(0.04)	2.86(0.81)	<.0001*
Hips	0.8(0.34)	1.16(0.38)	0.17
Knees	0.19(0.05)	0.54(0.12)	.01*
Lower Legs	0.50(0.19)	0.55(0.12)	0.16
Ankles	0.22(0.07)	0.76(0.25)	.005*
Feet	0.31(0.08)	0.57(0.23)	0.05
Overall Fatigue	1.41(0.35)	2.42(0.43)	0.30

Note. Bold font and * indicate significant values, α = 0.05. Abbreviations: EXO = with exoskeleton; NOEXO = without exoskeleton; SE = standard error.

In females, discomfort in the hands/wrists and overall fatigue were significantly higher in the NOEXO condition compared to the EXO condition. In males, more differences were observed: discomfort was significantly higher without the exoskeleton in the elbows/arms, hands/wrists, lower back, knees, and ankles. However, no significant difference was found in overall fatigue between conditions.

Regarding the second section of the questionnaire, 22 out of 30 participants rated the task as easy to perform with the exoskeleton, selecting scores above 7/10. Additionally, 19 participants gave scores above 7/10 for how comfortable it is to perform the task with the exoskeleton. A total of 25 out of 30 participants indicated a preference for performing the task with the exoskeleton, and 28 out of 30 participants reported that they would recommend the exoskeleton for similar load-carrying tasks.

### Heart rate

In females, statistical analysis revealed a significantly lower mean heart rate during the EXO condition (M = 96.20, SE = 3.96 bpm) compared to the NOEXO condition (M = 107.60, SE = 3.97 bpm), *p* = .002. Similarly, in males, a significantly lower mean heart rate was observed in the EXO condition (M = 90.71, SE = 5.15 bpm) compared to the NOEXO condition (M = 99.86, SE = 5.16 bpm), *p* < .0001. Resting heart rates prior to each experimental condition were not significantly different for either sex and were both significantly lower than the corresponding mean heart rates during activity (*p* < .0001; Females: M = 75.15, SE = 2.10 bpm; Males: M = 77.15, SE = 4.04 bpm).

## Discussion

This study evaluated the effects of a passive upper-body exoskeleton (CarrySuit^Ⓡ^) on physiological, biomechanical, and perceptual outcomes during stair ascent and descent while carrying a load. Despite growing interest in passive exoskeletons for manual material handling, little is known about their impact during stair negotiation. By comparing heart rate, muscle activity, joint motion, and perceived discomfort in 30 participants carrying a 12 kg load with and without the exoskeleton, the findings demonstrate that the CarrySuit^Ⓡ^ can reduce muscular and cardiovascular strain and improve user comfort without significantly altering natural movement patterns that are critical for safe stair mobility.

### Muscle activity and cardiovascular response

As expected, the most consistent finding across conditions and sexes was the reduced mean and peak activation of the biceps brachii when using the exoskeleton. This aligns with previous reports on the CarrySuit^Ⓡ^ during carrying tasks on flat surfaces [13,40], which also demonstrated an effective reduction of upper-limb muscular effort compared to carrying without the exoskeleton. Regarding the lower back, a reduced activation was evident for both sexes during stair ascent, but not in females during stair descent. This suggests that the CarrySuit^Ⓡ^’s effect on reducing lumbar load may be more consistent in males during stair negotiation. While lumbar activity remained stable in females during descent, this did not indicate added low back strain, highlighting a positive benefit of the device for load carriage during stair negotiation. These findings are consistent with earlier evaluations of the CarrySuit^Ⓡ^ on level ground (13), treadmill walking [41], and inclined surfaces (15), as well as with a study involving an active back-support exoskeleton [42].

In the upper leg, males exhibited increased vastus lateralis activation during stair ascent and a notable asymmetry during stair descent when using the exoskeleton, with approximately 20% greater activation on the right side. Given that all participants were right-handed, this asymmetry may reflect habitual limb dominance influencing stabilization strategies or movement patterns rather than random variability. These upper-leg alterations were not observed in females, suggesting potential sex-specific differences in muscle recruitment patterns or stabilization strategies when using the CarrySuit^Ⓡ^ during stair negotiation. These patterns are in line with prior research evaluating the CarrySuit^Ⓡ^ during carrying tasks on level surfaces [14,15]. In the lower leg, gastrocnemius medialis tended to be lower with the exoskeleton, particularly during stair descent, which contrast with reports of increased calf muscle demand in other back-support exoskeletons during industrial tasks [43,44]. This suggests that lower-limb muscle responses are influenced by specific design of the exoskeleton and the nature of the task. Notably, in the present study, gastrocnemius activation was asymmetric in females under both conditions, but the magnitude of asymmetry was reduced with the exoskeleton (~10%) compared to without it (~30%). Previous research has suggested that asymmetries exceeding 20% may indicate neuromuscular imbalance and could increase the risk of falling [45,46]. Therefore, the reduced asymmetry observed with the exoskeleton may reflect a more balanced muscle recruitment strategy during stair negotiation.

Finally, the reduction in muscle activity observed with the exoskeleton is supported by significantly lower heart rates in the EXO condition for both sexes, indicating reduced cardiovascular strain and overall physical effort during stair negotiation. Importantly, this reduction occurred despite the added weight of the CarrySuit^Ⓡ^, suggesting that the device’s ergonomic support more than offset its mass. These results reinforce the notion that the exoskeleton effectively reduces physiological load, even under complex, vertical movement tasks. This finding is also consistent with previous studies on carrying tasks with the CarrySuit^Ⓡ^ [13,15,41].

### Kinematics

Kinematic analysis revealed condition-dependent changes in sagittal plane joint angles. Notably, the EXO condition was associated with reduced joint ROM in several upper and lower body segments, particularly in males. Since the CarrySuit^Ⓡ^ is anchored at the pelvis and secured with straps around the trunk, it may contribute to maintaining a more neutral alignment of the pelvis and trunk during movement. Similar changes in pelvic and trunk positioning have been documented in gait studies involving backpack loads [47], as well as in previous assessments of the CarrySuit^Ⓡ^ on both level and inclined surfaces [14,15], particularly among male participants.

In females, reductions in ROM were limited to the neck and thorax, while increases were observed at the knees and hips during descent, and at the ankles, hips, and shoulders during ascent. This increased ROM at the knees and hips during descent may reflect a more dynamic or deeper movement strategy, which could demand greater postural control. Such compensatory control could explain the absence of reduced erector spinae activation observed in females during this phase. For males, ROM increases were confined to the ankles and knees during stair ascent.

Prior research has shown that carrying frontal loads on stairs tends to increase hip and knee ROM and reduce ankle dorsiflexion, potentially elevating biomechanical demand and fall risk [48,49]. In this study, while the EXO condition was associated with some increases in lower-limb ROM, particularly in females, ankle mobility was preserved, and exaggerated hip and knee angles were not substantially amplified. Importantly, the exoskeleton appeared to mitigate thoracic ROM during stair ascent, which contrasts with previous findings showing increased trunk tilt during loaded versus unloaded stair climbing [18,19,48]. This suggests a possible stabilizing effect of the exoskeleton on trunk posture. Overall, these findings indicate that the CarrySuit^Ⓡ^ does not restrict essential joint mobility and may help promote more stable movement patterns during stair negotiation, particularly given that no asymmetries in joint angles were observed with the exoskeleton. Moreover, although some reductions in joint ROM were observed, all values remained within functional and normative ranges [50,51], indicating that these changes are not restrictive or detrimental to task performance.

### Perceived discomfort and user feedback

Perceived discomfort and fatigue ratings further support the physiological findings. Female participants reported significantly less discomfort in the hands and wrists, along with lower overall fatigue when using the exoskeleton. Male participants experienced even broader relief, with reduced discomfort reported in multiple regions, including arms, lower back, knees, and ankles. These subjective outcomes are consistent with the observed reductions in muscle activation and with previous evaluations of the CarrySuit^Ⓡ^ [13,14,41]. This may reflect the exoskeleton’s effective redistribution of load, as contact forces are transferred to different body regions, thereby reducing localized muscular demand, such as in the biceps brachii and erector spinae. Notably, user satisfaction was high: over 80% of participants rated the device as easy and comfortable to use, and more than 90% indicated they would recommend it for similar load-carrying tasks. These findings highlight the exoskeleton’s potential for real-world acceptance and practical implementation in occupational settings.

### Study limitations

This study has several limitations. First, although stair tasks are highly relevant in occupational settings, the results may not be generalizable to other vertical or uneven terrains. Second, the standardized load of 12 kg may not capture the full range of load demands encountered in real-world job scenarios. Third, while a familiarization period was provided prior to testing, potential adaptation effects may still have influenced participant performance. Fourth, the participant sample consisted primarily of relatively young individuals, which may limit the applicability of the findings to a more age-diverse workforce. Fifth, all participants were university students, and data on physical activity levels or occupational background were not collected, which may limit the generalizability of the results to more diverse working populations. Finally, long-term use effects were not assessed. Nevertheless, the observed reductions in muscle activation and cardiovascular strain strongly support the ergonomic potential of the CarrySuit^Ⓡ^’s design. Future studies should investigate the cumulative impact of exoskeleton use over extended work durations and examine performance across a wider variety of environmental and task conditions.

## Conclusions

The present study evaluated the biomechanical and physiological effects of a passive upper-body exoskeleton (CarrySuit^Ⓡ^) during stair ascent and descent while carrying a 12 kg load. The findings indicate that the exoskeleton effectively reduced muscle activation in the biceps brachii and erector spinae during stair negotiation. However, this reduction in erector spinae activity was not observed in female participants during descent, although activation levels remained comparable to the condition without exoskeletal support. Lower-leg muscle activity during descent was reduced across all participants, while an increase in upper-leg muscle activation was observed exclusively in males during ascent. Side-to-side differences in lower-limb muscle activity were minimal with the exoskeleton, and no asymmetries were found in joint ROM. Reductions in joint movement amplitude were observed in several segments, particularly in males, with the most notable decreases at the shoulders, elbows, pelvis, hips, and thorax. In females, reductions were limited to the neck and thorax. In contrast, increases in ROM were noted in the ankles and knees in both sexes, and in the hips and shoulders among females. Overall, the CarrySuit^Ⓡ^ significantly reduced muscular and cardiovascular strain and improved user comfort during stair-based load carrying, although effects varied by sex and movement direction. Importantly, these benefits were achieved without compromising essential joint mobility, highlighting the exoskeleton’s potential as a supportive ergonomic intervention for occupational tasks involving stair negotiation. Notably, these positive outcomes were observed despite the exoskeleton’s additional 5.6 kg of mass, demonstrating a net physiological benefit even in demanding vertical tasks where added weight might otherwise be expected to increase strain. Future research should explore the long-term effects of exoskeleton use across more diverse occupational populations and environmental conditions.

## Supporting information

S1 Fig Phases of the stair ascent gait cycle. Illustration of the seven phases of stair ascent. The leading leg is shown in orange. This schematic supports the kinematic segmentation described in the Methods section.
(TIF)

## References

[1] Eurofound and International Labour Organization. Working conditions in a global perspective. Publications Office of the European Union, Luxembourg, and International Labour Organization, Geneva; 2019. 196 p. Available from: https://www.eurofound.europa.eu/publications/report/2019/working-conditions-in-a-global-perspective

[2] da Costa BR, Vieira ER. Risk factors for work-related musculoskeletal disorders: a systematic review of recent longitudinal studies. Am J Ind Med. 2010;53(3):285–323. doi: 10.1002/ajim.20750 19753591

[3] Farioli A, Mattioli S, Quaglieri A, Curti S, Violante FS, Coggon D. Musculoskeletal pain in Europe: the role of personal, occupational, and social risk factors. Scand J Work Environ Health. 2014;40(1):36–46. doi: 10.5271/sjweh.3381 24009006 PMC3964819

[4] Garcia M-G, Graf M, Läubli T. Lower limb pain among workers: a cross-sectional analysis of the fifth European Working Conditions Survey. Int Arch Occup Environ Health. 2017;90(7):575–85. doi: 10.1007/s00420-017-1220-4 28417255 PMC5934451

[5] Nieminen LK, Pyysalo LM, Kankaanpää MJ. Prognostic factors for pain chronicity in low back pain: a systematic review. Pain Rep. 2021;6(1):e919. doi: 10.1097/PR9.0000000000000919 33981936 PMC8108595

[6] Bevan S. Economic impact of musculoskeletal disorders (MSDs) on work in Europe. Best Pract Res Clin Rheumatol. 2015;29(3):356–73. doi: 10.1016/j.berh.2015.08.002 26612235

[7] Hulshof CTJ, Pega F, Neupane S, van der Molen HF, Colosio C, Daams JG, et al. The prevalence of occupational exposure to ergonomic risk factors: a systematic review and meta-analysis from the WHO/ILO Joint Estimates of the Work-related Burden of Disease and Injury. Environ Int. 2021;146:106157. doi: 10.1016/j.envint.2020.106157 33395953

[8] Espinoza MA, Bilbeny N, Abbott T, Carcamo C, Zitko P, Zamorano P, et al. Cost analysis of chronic pain due to musculoskeletal disorders in Chile. PLoS One. 2022;17(10):e0273667. doi: 10.1371/journal.pone.0273667 36301984 PMC9612497

[9] Zhou X, Zheng L. Model-based comparison of passive and active assistance designs in an occupational upper limb exoskeleton for overhead lifting. IISE Trans Occup Ergon Hum Factors. 2021;9(3–4):167–85. doi: 10.1080/24725838.2021.1954565 34254566 PMC8789934

[10] de Looze MP, Bosch T, Krause F, Stadler KS, O’Sullivan LW. Exoskeletons for industrial application and their potential effects on physical work load. Ergonomics. 2016;59(5):671–81. doi: 10.1080/00140139.2015.1081988 26444053

[11] Baltrusch SJ, van Dieen JH, van Bennekom CAM, Houdijk H. Testing an exoskeleton that helps workers with low-back pain: less discomfort with the passive SPEXOR trunk device. IEEE Robot Automat Mag. 2020;27(1):66–76. doi: 10.1109/mra.2019.2954160

[12] Goršič M, Novak VD. Effects of the Auxivo CarrySuit occupational exoskeleton when carrying front and side loads on a treadmill. J Biomech. 2023;156:111692. doi: 10.1016/j.jbiomech.2023.111692 37348177

[13] Garcia G, Arauz PG, Alvarez I, Encalada N, Vega S, Martin BJ. Impact of a passive upper-body exoskeleton on muscle activity, heart rate and discomfort during a carrying task. PLoS One. 2023;18(6):e0287588. doi: 10.1371/journal.pone.0287588 37352272 PMC10289366

[14] Garcia G, Arauz PG, Alvarez I, Encalada N, Vega S, Baldo M, et al. Effects of a passive upper-body exoskeleton on whole-body kinematics, leg muscle activity, and discomfort during a carrying task. PLoS One. 2024;19(7):e0304606. doi: 10.1371/journal.pone.0304606 38990910 PMC11238980

[15] Garcia G, Yañez R, Espoz M, Albuja C, Arauz PG, Martin BJ. Biomechanical and physiological effects of an upper-body exoskeleton during simulated load-carrying on an inclined surface. PLoS One. 2025;20(6):e0325230. doi: 10.1371/journal.pone.0325230 40460164 PMC12132972

[16] Lencioni T, Carpinella I, Rabuffetti M, Marzegan A, Ferrarin M. Human kinematic, kinetic and EMG data during different walking and stair ascending and descending tasks. Sci Data. 2019;6(1):309. doi: 10.1038/s41597-019-0323-z 31811148 PMC6897988

[17] Lencioni T, Piscosquito G, Rabuffetti M, Sipio ED, Diverio M, Moroni I, et al. Electromyographic and biomechanical analysis of step negotiation in Charcot Marie Tooth subjects whose level walk is not impaired. Gait Posture. 2018;62:497–504. doi: 10.1016/j.gaitpost.2018.04.014 29679921

[18] Wang J, Gillette JC. Carrying asymmetric loads during stair negotiation: loaded limb stance vs. unloaded limb stance. Gait Posture. 2018;64:213–9. doi: 10.1016/j.gaitpost.2018.06.113 29933184

[19] Wang J, Gillette J. Carrying asymmetric loads during stair negotiation. Gait Posture. 2017;53:67–72. doi: 10.1016/j.gaitpost.2017.01.006 28113074

[20] Raghuraman RN, Barbieri DF, Aviles J, Srinivasan D. Age and gender differences in the perception and use of soft vs. rigid exoskeletons for manual material handling. Ergonomics. 2024;67(11):1453–70. doi: 10.1080/00140139.2024.2338268 38613461

[21] Hermens HJ, Freriks B. SENIAM. European Recommendations for Surface ElectroMyoGraphy. Enschede, the Netherlands: Roessingh Research and Development; 1999.

[22] Taylor EW, Ugbolue UC, Gao Y, Gu Y, Baker JS, Dutheil F. Erector spinae muscle activation during forward movement in individuals with or without chronic lower back pain: a systematic review and meta-analysis. Arch Rehabil Res Clin Transl. 2023;5(3):100280. doi: 10.1016/j.arrct.2023.100280 37744192 PMC10517367

[23] Merletti R, Cerone GL. Tutorial. Surface EMG detection, conditioning and pre-processing: Best practices. J Electromyogr Kinesiol. 2020;54:102440. doi: 10.1016/j.jelekin.2020.102440 32763743

[24] Konrad P. The ABC of EMG A practical introduction to kinesiological electromyography. 1.4. Scottsdale: Noraxon U.S.A., Inc.; 2006. Available from: www.noraxon.com

[25] Dennerlein JT, Johnson PW. Changes in upper extremity biomechanics across different mouse positions in a computer workstation. Ergonomics. 2006;49(14):1456–69. doi: 10.1080/00140130600811620 17028089

[26] Arauz PG, García M-G, Velez M, León C, Velez F, Martin B. Does treadmill workstation use affect user’s kinematic gait symmetry? PLoS One. 2021;16(12):e0261140. doi: 10.1371/journal.pone.0261140 34905578 PMC8670710

[27] Arauz PG, Garcia M-G, Chiriboga P, Taco-Vasquez S, Klaic D, Verdesoto E, et al. Spine and lower body symmetry during treadmill walking in healthy individuals-In-vivo 3-dimensional kinematic analysis. PLoS One. 2022;17(10):e0275174. doi: 10.1371/journal.pone.0275174 36201499 PMC9536630

[28] Grood ES, Suntay WJ. A joint coordinate system for the clinical description of three-dimensional motions: application to the knee. J Biomech Eng. 1983;105(2):136–44. doi: 10.1115/1.3138397 6865355

[29] Arauz P, Peng Y, MacAuliffe J, Kwon Y-M. In-vivo 3-Dimensional gait symmetry analysis in patients with bilateral total hip arthroplasty. J Biomech. 2018;77:131–7. doi: 10.1016/j.jbiomech.2018.07.013 30037578

[30] Kuorinka I, Jonsson B, Kilbom A, Vinterberg H, Biering-Sørensen F, Andersson G, et al. Standardised Nordic questionnaires for the analysis of musculoskeletal symptoms. Appl Ergon. 1987;18(3):233–7. doi: 10.1016/0003-6870(87)90010-x 15676628

[31] Garcia M-G, Läubli T, Martin BJ. Long-term muscle fatigue after standing work. Hum Factors. 2015;57(7):1162–73. doi: 10.1177/0018720815590293 26048874

[32] Garcia M-G, Tapia P, Läubli T, Martin BJ. Physiological and neuromotor changes induced by two different stand-walk-sit work rotations. Ergonomics. 2020;63(2):163–74. doi: 10.1080/00140139.2019.1677949 31594482

[33] Garcia M-G, Läubli T, Martin BJ. Muscular and vascular issues induced by prolonged standing with different work-rest cycles with active or passive breaks. Hum Factors. 2018;60(6):806–21. doi: 10.1177/0018720818769261 29648891

[34] Talib I, Sundaraj K, Lam CK, Sundaraj S. A systematic review of muscle activity assessment of the biceps brachii muscle using mechanomyography. J Musculoskelet Neuronal Interact. 2018;18(4):446–62. 30511949 PMC6313049

[35] Hébert-Losier K, Holmberg H-C. Knee angle-specific MVIC for triceps surae EMG signal normalization in weight and non weight-bearing conditions. J Electromyogr Kinesiol. 2013;23(4):916–23. doi: 10.1016/j.jelekin.2013.03.012 23639755

[36] Kushion D. EMG activation of the vastus medialis oblique and vastus lateralis during four rehabilitative exercises. TOREHJ. 2012;5(1):1–7. doi: 10.2174/1874943701205010001

[37] Biviá-Roig G, Lisón JF, Sánchez-Zuriaga D. Determining the optimal maximal and submaximal voluntary contraction tests for normalizing the erector spinae muscles. PeerJ. 2019;7:e7824. doi: 10.7717/peerj.7824 31637121 PMC6802582

[38] Tippey KG, Longnecker MT. An ad hoc method for computing pseudo-effect size for mixed models. South Central SAS Users Group; 2016.

[39] Lakens D. Calculating and reporting effect sizes to facilitate cumulative science: a practical primer for t-tests and ANOVAs. Front Psychol. 2013;4:1–12. 24324449 10.3389/fpsyg.2013.00863 PMC3840331

[40] Garcia G, Yañez R, Espoz M, Albuja C, Arauz P, Martin BJ. Influence of an upper-body exoskeleton on kinematics, heart rate, and muscle activity while walking on an inclined surface. International Ergonomics Association (IEA) 2024 Congress. Seoul: Springer; 2024.

[41] Goršič M, Novak VD. Effects of the Auxivo CarrySuit occupational exoskeleton when carrying front and side loads on a treadmill. J Biomech. 2023;156:111692. doi: 10.1016/j.jbiomech.2023.111692 37348177

[42] Poliero T, Lazzaroni M, Toxiri S, Di Natali C, Caldwell DG, Ortiz J. Applicability of an active back-support exoskeleton to carrying activities. Front Robot AI. 2020;7:579963. doi: 10.3389/frobt.2020.579963 33501340 PMC7805869

[43] Luger T, Bär M, Seibt R, Rieger MA, Steinhilber B. Using a back exoskeleton during industrial and functional tasks-effects on muscle activity, posture, performance, usability, and wearer discomfort in a laboratory trial. Hum Factors. 2023;65(1):5–21. doi: 10.1177/00187208211007267 33861139 PMC9846378

[44] Riemer J, Wischniewski S, Jaitner T. Quantifying the biomechanical effects of back-support exoskeletons on work movements using statistical parametric mapping. J Safety Res. 2024;91:492–504. doi: 10.1016/j.jsr.2024.09.010 39998548

[45] Perry MC, Carville SF, Smith ICH, Rutherford OM, Newham DJ. Strength, power output and symmetry of leg muscles: effect of age and history of falling. Eur J Appl Physiol. 2007;100(5):553–61. doi: 10.1007/s00421-006-0247-0 16847676

[46] Laroche DP, Cook SB, Mackala K. Strength asymmetry increases gait asymmetry and variability in older women. Med Sci Sports Exerc. 2012;44(11):2172–81. doi: 10.1249/MSS.0b013e31825e1d31 22617401 PMC3463648

[47] Genitrini M, Dotti F, Bianca E, Ferri A. Impact of backpacks on ergonomics: biomechanical and physiological effects: a narrative review. Int J Environ Res Public Health. 2022;19(11):6737. doi: 10.3390/ijerph19116737 35682317 PMC9180465

[48] Smadi O, Abu Alim MA, Masad IS, Almashaqbeh S. The influence of carrying anterior load on the sagittal and frontal plane kinematics of lower extremities during stair ascending. J Biomed Phys Eng. 2021;11(1):93–102. doi: 10.31661/jbpe.v0i0.2007-1143 33564644 PMC7859378

[49] Tseng H-Y, Liu B-S. Effects of load carrying methods and stair slopes on physiological response and postures during stairs ascending and descending. Ind Health. 2011;49(1):30–6. doi: 10.2486/indhealth.ms1100 20823636

[50] Protopapadaki A, Drechsler WI, Cramp MC, Coutts FJ, Scott OM. Hip, knee, ankle kinematics and kinetics during stair ascent and descent in healthy young individuals. Clin Biomech (Bristol). 2007;22(2):203–10. doi: 10.1016/j.clinbiomech.2006.09.010 17126461

[51] Lin H-C, Lu T-W, Hsu H-C. Three-dimensional analysis of kinematic and kinetic coordination of the lower limb joints during stair ascent and descent. Biomed Eng Appl Basis Commun. 2004;16(02):101–8. doi: 10.4015/s1016237204000153

